# Combination of Multifocal Electroretinogram and Spectral-Domain OCT Can Increase Diagnostic Efficacy of Parkinson's Disease

**DOI:** 10.1155/2018/4163239

**Published:** 2018-03-01

**Authors:** Jiang Huang, Yi Li, Jianjiang Xiao, Qin Zhang, Guoxu Xu, Guanhui Wu, Tong Liu, Weifeng Luo

**Affiliations:** ^1^Department of Ophthalmology, Second Affiliated Hospital of Soochow University, Suzhou, China; ^2^Department of Ophthalmology, Huashan Hospital North, Fudan University, Shanghai, China; ^3^Department of Neurology, Second Affiliated Hospital of Soochow University, Suzhou, China; ^4^Institute of Neuroscience, Soochow University, Suzhou, China

## Abstract

**Background:**

The retinal changes have been identified in morphology and function in Parkinson's disease (PD). However, the controversial results suggest that it is incredible that only using a single method for testing retinal change to evaluate Parkinson's disease. The aim of this study was to assess retinal changes and increase the diagnostic efficacy of Parkinson's disease with a combination of multifocal electroretinogram (mf-ERG) and spectral domain optical coherence tomography (SD-OCT) examinations.

**Method:**

Fifty-three PD patients and forty-one healthy controls were enrolled. Subjects were assessed for retinal function using mf-ERG and retinal structure using SD-OCT.

**Results:**

The PD patients had a significantly decreased amplitude density of P1 and a delayed implicit time of P1 in some regions. The macular retinal thickness, macular volume, and average RNFL thickness were decreased in PD. The AUC of a single parameter of either retinal function or structure was low. Both of them were higher in diagnostic value to discriminate PD patients.

**Conclusion:**

The amplitude density of P1 combined with macular volume can get a high diagnostic efficacy to discriminate between participants with or without PD. It indicates that a combination of mf-ERG and SD-OCT provides a good clinical biomarker for diagnosis of PD.

## 1. Introduction

Parkinson's disease (PD) is the second most common neurodegenerative disorder affecting middle-aged and elderly people in the developed world [1, 2]. The clinical manifestations of Parkinson's disease are multisystem disorders with a wide variety of motor and nonmotor features [3, 4]. And the nonmotor aspects include mood disturbance, sleep disorder, cognitive decline dementia, autonomic failure, and vision dysfunction [5, 6]. Many PD patients have vision symptoms when examined [7], yet the vision-related problem of PD still remains under-recognized and less understood.

It is known that PD can cause neural impairment outside the central nervous system even before damage to the basal ganglia. Vision deficits of PD are common including visual acuity, contrast sensitivity, ocular movement, color perception, and other damages in the vision system [6, 7]. Lots of studies [8–10] have demonstrated that retina, especially the fovea, where dopaminergic amacrine cells have concentrated in, is the vulnerable site of vision function in PD.

Multifocal electroretinogram (mf-ERG) is a sensitive and specific method for monitoring the functional changes of the posterior retina, especially in the annular zone surrounding the fovea [11, 12]. Previous studies [13–15] have shown that the mf-ERG test can reflect the function of fovea in several retinal diseases, such as age-related macular degeneration, diabetic macular edema, Best's disease, and so on. Few studies [16, 17] have shown that mf-ERG values revealed decreased foveal electrical activity in PD patients.

Spectral-domain optical coherence tomography (SD-OCT) is a new, advanced, noninvasive technology, which can provide cross-sectional images of the retina and optic in a rapid, objective, reproducible manner for evaluation of the thickness of macular and retinal nerve fiber layer (RNFL) [18, 19]. Most studies [16, 20, 21] suggested the thinning of macular thickness and the loss of RNFL in patients with PD; however, some studies [22, 23] found neither a reduction in macular thickness nor the loss of RNFL.

These conclusions show that the retinal function and structure have changed in PD patients, and it may prove these retinal monitor techniques will be useful potential biomarkers for diagnosis or assessing disease progression in PD. However, studies with controversial results also suggest that it is incredible that only using a single method for testing retinal structure or function to evaluate PD.

To date, there are no studies of correlations of mf-ERG and SD-OCT in the macular function and structure in large participants. In this study, we combined mf-ERG with SD-OCT test in PD patients and healthy control subjects, and we analyzed the alterations in functional and structural changes in PD and the association between these changes in the diagnostic yield of PD.

## 2. Method

This was a cross-sectional study and was performed according to the principles outlined in the Declaration of Helsinki and was approved by the Institutional Review Board of the second affiliated Hospital of Soochow University. All participants gave written informed consent prior to study inclusion.

### 2.1. Participants

Patients with idiopathic PD and healthy controls (HC) participants were enrolled in the study. All patients were prospectively recruited from the local neurologic department and underwent a complete neurologic examination. The severity of the disease was described using Hoehn and Yahr scale and unified Parkinson disease rating scale III (UPDRS III). The Ophthalmic examination included best-corrected visual acuity (BCVA), intraocular pressure (IOP), visual field using Octopus instrument, slit-lamp examination, dilated ophthalmoscopy, fundus photography, SD-OCT, and mf-ERG examination. The diagnosis of idiopathic PD was confirmed by the treating neurologist based on the United Kingdom Brain Bank criteria for the clinical diagnosis of idiopathic PD [24].

The exclusion criteria included patients with diabetes mellitus, poor sitting stability, recognition disorder, and history of severe visual loss including cataract, glaucoma, age-related macular degeneration, hypermyopia (refractive diopter >−4.0D), and any ocular surgery.

### 2.2. mf-ERG Recording

Mf-ERG test was recorded according to the International Society for Clinical Electrophysiology of Vision guidelines [25] using a visually evoked test system (VETS V8.1; GOTEC, Chongqing). Pupils were dilated (≥7 mm) using 1.0% tropicamide and 2.5% phenylephrine. A Burian-Allen contact lens electrode was used, which was placed on the anesthetized (0.4% oxybuprocaine hydrochloride) cornea. A ground electrode was clipped to the right earlobe, and the electrode impedance was maintained below 5 kΩ. The patients were positioned at a distance of 33 cm from the stimulus monitor. The stimulus was presented on a 19-inch CRT. The mf-ERG system was used with a scaled 103-hexagon stimulus element displayed on a 19-inch CRT with a frame rate of 75 Hz. The hexagons were modulated between white (200 cd/m^2^) and black (<2 cd/m^2^) according to an m-sequence during the 8-minute recording sessions. The stimulus array was positioned on the retina at approximately 45° and centered on the fovea. Recordings were collected in sixteen segments of approximately 25 seconds. Fixation was controlled using an “x” target in the center of the stimulus. The contaminated segments were discarded and reevaluated.

### 2.3. SD-OCT Examination

Pupils were dilated (≥7 mm) using 1.0% tropicamide and 2.5% phenylephrine. SD-OCT was performed using Cirrus HD-OCT Model 4000 (Carl Zeiss Meditec Inc.). The macular zone including of the macular retinal thickness (MRT), central foveal thickness, and macular volume (MV) was assessed using a Macular Cube 512 × 128 scanning. The RNFL thickness was assessed using an Optic Disc Cube 200 × 200 scanning.

### 2.4. Data Analysis

Statistical analysis was performed using SPSS 20.0. Data were presented as mean ± SD as appropriate. Initial data analysis consisted of comparing the mean age between groups through a 2-tailed *t*-test. The parameters of mf-ERG and SD-OCT were evaluated using the Mann–Whitney *U* test. The Pearson chi-square test was employed for comparison of the frequencies. The correlation of the structural and functional changes in retina was evaluated using the Pearson correlation coefficient. A *p* value less than 0.05 was considered significant.

The mf-ERG test was analyzed to determine the amplitude density (AD) and implicit time (IT). The IT of P1 (first positive peak) and amplitudes (N1-P1) were analyzed for the specified areas in five rings including ring1, ring2, ring3, ring4, and ring5. The five rings represent the summed responses from five adjacent concentric ring-shaped areas.

The analysis of parameters of SD-OCT consisted of macular retinal thickness (MRT), central foveal thickness (CFT), macular volume (MV), and retinal nerve fiber layer (RNFL) thickness. The RNFL thickness was analyzed with the average RNFL and four quadrants including temporal quadrant thickness, superior quadrant thickness, nasal quadrant thickness, and inferior quadrant thickness.

## 3. Results

### 3.1. Basic Demographics Analysis

The participants' basic demographics are shown in Table 1. The two groups did not differ significantly in age, sex, intraocular pressure (IOP), or best-corrected visual acuity (BCVA). The PD group included male and female with a mean age of 61.79 ± 9.89 years (range: 32–81 years). The mean duration of PD was 67.92 ± 45.52 months (range: 10–204 months) with a median of 60 months since diagnosis.

There was a significant difference in the mean deviation (MD) of the visual field between the HC group and the PD group (*u*=−4.060,  *p* ≤ 0.001). The basic demographics of parameters including age, sex, BCVA, and IOP had no difference between the two groups.

### 3.2. The mf-ERG Examination Results

The amplitude density (AD) of P1 differed significantly in ring1 and ring2 between the HC group and PD patients group (*p* ≤ 0.001). Compared with the HC group, the IT of P1 in ring1, ring2, and ring3 was significantly prolonged in the PD group (*p* ≤ 0.001). The AD and IT of N1 wave did not differ significantly among the rings (Table 2).

### 3.3. The SD-OCT Examination Results

The SD-OCT examinations results are summarized in Table 3. The macular retinal thickness (MRT) and macular volume (MV) were decreased in the PD group compared with healthy controls (*p*=0.027,  0.001, resp.). The average of RNFL thickness and inferior quadrant thickness was obviously thinning in PD patients compared with the HC group (*p*=0.008,  0.004, resp.). However, there was no significant difference in the other three quadrants of RNFL thickness between the two groups.

### 3.4. Correlation of Different Examination Results

The AD of P1 in ring1 was negatively correlated with the MD of the visual field (*p*=0.008) and positively correlated with MV (*p*=0.005). The AD of P1 in ring2 was negatively correlated with the MD of the visual field (*p*=0.003) and positively correlated with MV (*p*=0.012). The IT of P1 in ring3 was positively correlated with the MD of visual field (*p*=0.015) and average RNFL thickness (*p*=0.027) (Table 4).

### 3.5. The ROC Curve Analyses of the Different Examinations Results

**Figure 1:**
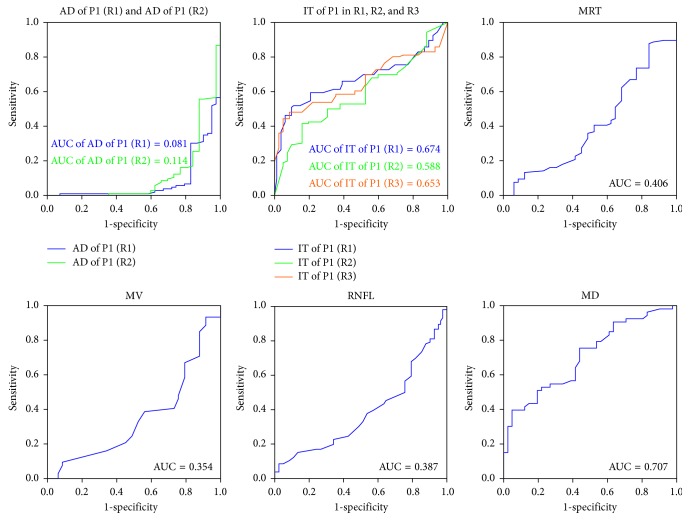
ROC curves of the single parameter of visual function or structure for discriminating Parkinson's disease. AD = amplitude density; IT = implicit time; R1 = ring1; R2 = ring2; R3 = ring3; MD = mean deviation of visual field; MRT = macular retinal thickness; MV = macular volume; RNFL = retinal nerve fiber layer.

ROC (receiver operating characteristic) curves of single parameter were plotted in Figure 1. The AUC (area under curve) of the AD of P1 in ring1 and ring2 were 0.081 and 0.114, respectively. The IT of P1 in ring1, ring2, and ring3 to detect PD diagnosis was 0.674, 0.588, and 0.653, respectively. The MRT, MV, and RNFL revealed AUC of 0.406, 0.354, and 0.387, respectively. The MD of the visual field to detect PD diagnosis was 0.709.

**Figure 2:**
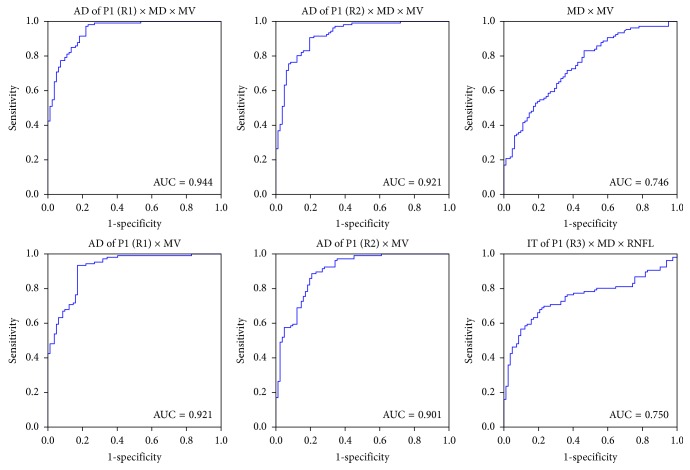
ROC curves of different combinations of visual function and structure parameters for discriminating Parkinson's disease. AD = amplitude density; IT = implicit time; R1 = ring1; R2 = ring2; R3 = ring3; MD = mean deviation of visual field, MRT = macular retinal thickness; MV = macular volume; RNFL = retinal nerve fiber layer.

ROC curves of the combinations of these parameters were plotted in Figure 2. The AUC (area under curve) of a combination of the AD of P1 in ring1, the MD and MV, to detect PD diagnosis, was 0.944. A combination of the AD of P1 in ring2, the MD and the MV, revealed AUC of 0.921. A combination of the AD of P1 in ring1 and the MV revealed AUC of 0.921. The AUC of the MD combined with the MV, the AD of P1 in ring1 combined with the MV, and the AD of P1 in ring2 combined with the MV were 0.746, 0.922, and 0.901, respectively. A combination of the IT of P1 in ring3, the MD, and the average RNFL thickness revealed AUC of 0.750 (Figure 2).

## 4. Discussion

Because of the relatively early stage of disease of recruited PD patients in our study, the visual acuity was normal and had no significant difference compared with the HC group. However, the abnormalities of the MD of the visual field were observed with the significant difference. Lots of studies [6, 23, 26] suggested that the MD of the visual field was a sensitive parameter of the visual function in patients with PD. The visual field test reflects the function of retinal ganglion cells, which have axons that project via the optic nerve to diverse targets in the brain.

Many previous studies [12, 18, 19] on the vision function of PD patients have used visual evoked potential (VEP). However, the VEP monitors the integrity of the entire visual pathway from the fovea to the visual cortex, so it can't be an accurate response to the fovea function. The mf-ERG test is specific and sensitive for reflecting the function of the fovea, where the density of the ganglion cell is much higher than other zones of the retina. To date, few studies [16, 17] have focused on PD with the mf-ERG test. In our study, we found that the amplitude of P1 was decreased and the IT of P1 was prolonged in ring1 and ring2 significantly compared with the HC group. The result was consistent with that of study of Kaur et al. [16], which showed that mf-ERG in central 2° revealed the reduced foveal activity in PD patients.

In recent years, since the application of SD-OCT instrument for measurement of the retinal thickness in patients with PD, lots of studies [16–23] focused on the structural changes in the retina of PD. However, the results had no agreement in conclusions. It may be because of the recruited patients with different disease stages or the difference of diversity of OCT apparatus.

The macular thickness analysis in our study revealed the thinning change in macular retinal thickness, macular volume, and average RNFL thickness of patients with PD compared with the HC group. Many studies [16, 20] confirmed this. However, some studies [23–29] had conflicting results. There are some possible reasons for it. First, in our study, the mean of Hoehn–Yahr scales is 1.92 ± 0.54 and UPDRS III scales is 37.15 ± 16.61, which suggested that the disease stage of recruited PD patients was early, but the motor manifestation was serious, so the change of thickness of RNFL may be obvious. Second, the age of recruited patients and the HC participants in our study was much younger than that of in previous studies [23, 29]. However, the RNFL is negatively correlated with age, making difficulties in detection of the subtle differences of RNFL in the older participants. So, when the patients are much older, it may be not easy to find the thinning changes caused by PD.

All of these reasons suggested that it was incredible to use a single method to evaluate the retinal change in patients with PD. Some studies confirmed that it was vital to combine functional and structural changes to evaluate the PD. Miri et al. [29] and Altintaş et al. [30] reported a near negative correlation between the total MV and P100 latency in PD and found that the pattern VEP combined with retinal foveal thickness had a high diagnostic yield for PD. Garcia-Martin et al. [21] reported that both of the AD of P-ERG and the thickness of macular and RNFL were decreased in PD patients.

These studies demonstrated that the retina of patients with PD had changed in both of morphology (retinal thickness) and retinal function including visual field, VEP test, and pattern ERG. We assumed that it could find more clinical diagnostic values by integrating the test parameters. So, the main aim of our study was to evaluate retinal changes and increase the diagnostic yield of PD with a combination of mf-ERG and SD-OCT examinations.

We found an important correlation between macular morphology and function in patients with PD. The AD of P1 in some regions was negatively correlated with the MD of the visual field and positively correlated with MV. And the IT of P1 in some regions was positively correlated with the MD. That means there is a strong internal consistency between retinal structure and retinal function.

Further analysis of the ROC curve of multiparameter can lead to more information on clinical diagnostic value. This study indicates that the ROC curve for a combination of retinal structure and retinal function can much better discriminate between participants with PD and healthy controls than that for a single parameter. It demonstrated that the AD of P1 in ring1 combined with the MD and the MV had the highest diagnostic yield of PD. However, the less the parameters selected, the higher the diagnostic effectiveness. In our study, the AUC of the AD of P1 in ring2 combined with the MD of the visual field and the MV equaled that of the AD of P1 in ring1 combined with the MV. On the contrary, a combination of the IT of P1 in ring3 and the MD and the RNFL revealed a lower AUC. The AD of P1 in ring2 combined with the MV actually had a higher AUC, although there were only two parameters selected. It demonstrated that the AD of P1 combined with the MV could get a high diagnostic yield to discriminate between participants with or without PD.

By analysis of ROC curve, this study found that a single parameter of either function or structure revealed lower AUC significantly than that of the combined parameters. So, it indicates that a combination of mf-ERG and SD-OCT provides a good clinical biomarker for diagnosis of PD.

## Figures and Tables

**Table 1:** Demographics, disease characteristics, and visual field of all participants.

	HC	PD	*p*
Number of subjects	*n*=41	*n*=53	
Age (years)	62.29 ± 9.74	61.79 ± 9.89	0.807^a^
Male/female	24/17	35/18	0.456^b^
PD duration (months)	—	67.92 ± 45.52	—
BCVA	1.01 ± 0.03	0.99 ± 0.04	0.096^c^
MD of visual field (dB)	2.94 ± 3.25	7.00 ± 6.39	≤0.001^c^^∗∗^
IOP (mmHg)	14.79 ± 2.93	14.19 ± 2.97	0.338^c^
Hoehn and Yahr scale	—	1.92 ± 0.54	—
UPDRS III	—	37.15 ± 16.61	—

Values are expressed as mean ± SD (unless otherwise stated). Statistical tests: ^a^*t*-test; ^b^Pearson's *χ*^2^ test; ^c^Mann–Whitney *U* test. ^∗∗^*p* < 0.01. BCVA, best-corrected visual acuity; MD, mean deviation (dB); IOP, intraocular pressure (mmHg); UPDRS III, Unified Parkinson Disease Rating Scale III.

**Table 2:** Retinal function analysis of PD patients and healthy subjects using mf-ERG.

	HC	PD	*p*
Number of eyes tested	*n*=82	*n*=106	
AD of P1 in ring1	139.43 ± 16.92	100.75 ± 21.13	≤0.001^∗∗^
AD of P1 in ring2	36.93 ± 9.09	21.83 ± 7.49	≤0.001^∗∗^
AD of P1 in ring3	15.79 ± 4.26	15.72 ± 4.00	0.692
AD of P1 in ring4	13.79 ± 3.23	13.30 ± 4.04	0.079
AD of P1 in ring5	10.08 ± 3.61	9.30 ± 1.74	0.062
IT of P1 in ring1	38.34 ± 4.85	42.72 ± 7.66	≤0.001^∗∗^
IT of P1 in ring2	39.47 ± 6.21	42.72 ± 7.66	0.039^∗^
IT of P1 in ring3	37.26 ± 4.42	41.09 ± 7.68	≤0.001^∗∗^
IT of P1 in ring4	41.14 ± 4.53	41.67 ± 8.51	0.656
IT of P1 in ring5	43.24 ± 6.65	41.72 ± 8.14	0.159
AD of N1 in ring1	0.48 ± 0.22	0.43 ± 0.13	0.071
AD of N1 in ring2	0.27 ± 0.05	0.26 ± 0.07	0.121
AD of N1 in ring3	0.19 ± 0.13	0.16 ± 0.08	0.395
AD of N1 in ring4	0.20 ± 0.09	0.19 ± 0.07	0.872
AD of N1 in ring5	0.17 ± 0.10	0.16 ± 0.08	0.910
IT of N1 in ring1	19.76 ± 3.21	20.47 ± 4.67	0.149
IT of N1 in ring2	20.14 ± 5.41	21.94 ± 9.03	0.135
IT of N1 in ring3	23.06 ± 3.72	22.13 ± 6.58	0.230
IT of N1 in ring4	24.30 ± 7.06	23.03 ± 6.50	0.208
IT of N1 in ring5	21.61 ± 5.84	22.44 ± 6.58	0.675

Values are expressed as mean ± SD. Statistical tests: Mann–Whitney *U* test. AD = amplitude density (nV/deg^2^); IT = implicit time (ms). ^∗^*p* < 0.05; ^∗∗^*p* < 0.01.

**Table 3:** Macular thickness, macular volume, and RNFL thickness analysis using SD-OCT.

	HC	PD	*p*
Number of eyes	82	106	
Macular thickness			
Macular retinal thickness (microns ± SD)	269.93 ± 22.56	262.15 ± 25.80	0.027^∗^
Central foveal thickness (microns ± SD)	239.68 ± 40.17	231.21 ± 49.28	0.100
Macular volume (mm^3^)	9.87 ± 0.68	9.51 ± 0.85	0.001^∗∗^
RNFL thickness (microns ± SD)			
Average	92.72 ± 11.50	88.93 ± 18.79	0.008^∗∗^
Temporal quadrant thickness	68.23 ± 14.26	66.14 ± 18.26	0.117
Nasal quadrant thickness	65.96 ± 12.51	66.25 ± 20.53	0.347
Superior quadrant thickness	113.20 ± 22.53	111.27 ± 23.57	0.532
Inferior quadrant thickness	123.45 ± 25.93	113.08 ± 34.00	0.004^∗∗^

Values are expressed as mean ± SD. Statistical tests: Mann–Whitney *U* test. RNFL = retinal nerve fiber layer. ^∗^*p* < 0.05; ^∗∗^*p* < 0.01.

**Table 4:** Correlation between visual functional parameters and structural parameters.

	MD	MRT	MV	Average RNFL
AD of P1 (R1)	(−0.191, 0.008)^∗∗^	(0.193, 0.008)	(0.204, 0.005)^∗∗^	(0.081, 0.267)
AD of P1 (R2)	(−0.214, 0.003)^∗∗^	(0.139, 0.057)	(0.184, 0.012)^∗^	(0.081, 0.267)
IT of P1 (R1)	(0.096, 0.190)	(−0.083, 0.255)	(−0.023, 0.749)	(−0.074, 0.311)
IT of P1 (R2)	(0.072, 0.328)	(−0.002, 0.973)	(−0.027, 0.716)	(−0.008, 0.918)
IT of P1 (R3)	(0.178, 0.015)^∗^	(−0.102, 0.163)	(−0.010, 0.895)	(0.161, 0.027)^∗^

Statistical tests: Pearson's correlation coefficient. AD = amplitude density (nV/deg^2^); IT = implicit time (ms); MD = mean deviation; MRT = macular retinal thickness; MV = macular volume; RNFL = retinal nerve fiber layer ^∗^*p* < 0.05; ^∗∗^*p* < 0.01.
